# Perspective—Assessing Electrochemical, Aptamer-Based Sensors for Dynamic Monitoring of Cellular Signaling

**DOI:** 10.1149/2754-2726/ad15a1

**Published:** 2023-12-27

**Authors:** Celeste R. Rousseau, Hope Kumakli, Ryan J. White

**Affiliations:** 1 Department of Chemistry, University of Cincinnati, Cincinnati, Ohio 45221, United States of America; 2 Department of Electrical Engineering and Computer Science, University of Cincinnati, Cincinnati, Ohio 45221, United States of America

**Keywords:** aptamer, electrochemical aptamer-based sensors, cell signaling, electrochemistry

## Abstract

Electrochemical, aptamer-based (E-AB) sensors provide a generalizable strategy to quantitatively detect a variety of targets including small molecules and proteins. The key signaling attributes of E-AB sensors (sensitivity, selectivity, specificity, and reagentless and dynamic sensing ability) make them well suited to monitor dynamic processes in complex environments. A key bioanalytical challenge that could benefit from the detection capabilities of E-AB sensors is that of cell signaling, which involves the release of molecular messengers into the extracellular space. Here, we provide a perspective on why E-AB sensors are suited for this measurement, sensor requirements, and pioneering examples of cellular signaling measurements.

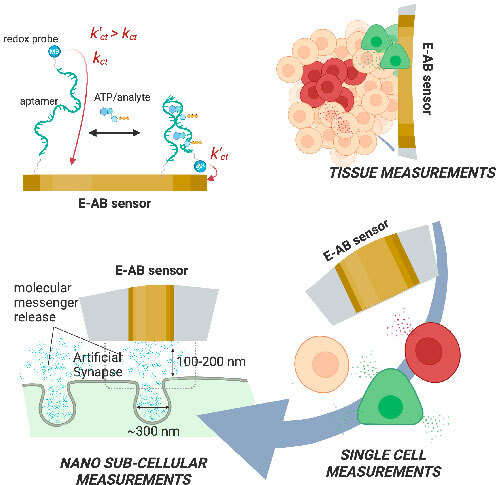

## Highlights

- Discussion of applications of electrochemical aptamer-based sensors.
- Overview of analytical challenges associated with use of aptamer-based sensors for real time cell metabolism measurements.
- Discussion of spatial resolution, electrode size requirement and dynamic range requirement of sensors for dynamic cellular signaling.
- Review and discussion of published work describing the current state of using electrochemical, aptamer-based sensors for measuring cellular signaling.

Cellular communication via the release of chemical messengers is a vital aspect of whole-organism function. The cross communication between cells, tissues, and organs is essential in, for example, neural, immune, and metabolic processes.^1^ Whether for hormonal, neuro- or other transmitter, or cytokine release, tight control over the extracellular concentrations of signaling molecules and biomolecules impacts cellular function and regulation of cellular pathways. The range of biological functions that cellular signaling controls and responds to is large and thus the range of concentrations, temporal dynamics, and chemical identities involved are extensive. This, from an analytical measurement scientist perspective, opens a wide parameter space in which molecules need to be detected. There are many analytical tools to monitor various aspects of cell signaling and the extracellular space ranging from mass spectrometry,^2–4^ microdialysis,^5,6^ to electrophysiological^7–10^ and electrochemical methods.^11–14^

Electrochemical techniques such as amperometry and fast scan cyclic voltammetry (FSCV) at carbon fiber microelectrodes have been employed to study the cellular release of signaling molecules from various cell lines, tissue, and in vivo.^15–18^ The measurement relies on the ability of small electrodes to make rapid and sensitive measurements of electroactive molecules. Examples include amperometric measurements of dopamine released from neurons in zeptomole quantities^16^ and use of voltametric techniques such as FSCV to quantify and provide information on the identity of various redox-active neurochemicals in immortalized cell lines, as well as in vivo.^17,18^ The key is that small electrodes enable rapid measurements (ms) due to small surface area and thus small RC time constants.

Enzymatic biosensors on microelectrodes expand detection capabilities to non-electrochemically active analytes. The operation of enzymatic biosensors is based on the interaction of an enzyme with a particular substrate, which results in a quantifiable signal related to the concentration of the analyte.^19^ An example of the use of small scale, enzymatic sensors is the development of enzyme-modified carbon fiber microelectrodes to quantify brain glucose events with sub-second temporal resolution.^20^ However, despite their applications, enzymatic biosensors still have challenges in practical implementation, including substrate interference, enzyme stability, sensor reproducibility, mass transfer limitations, and the ultimate limited availability of redox active enzymes suitable.

**Figure 1.**
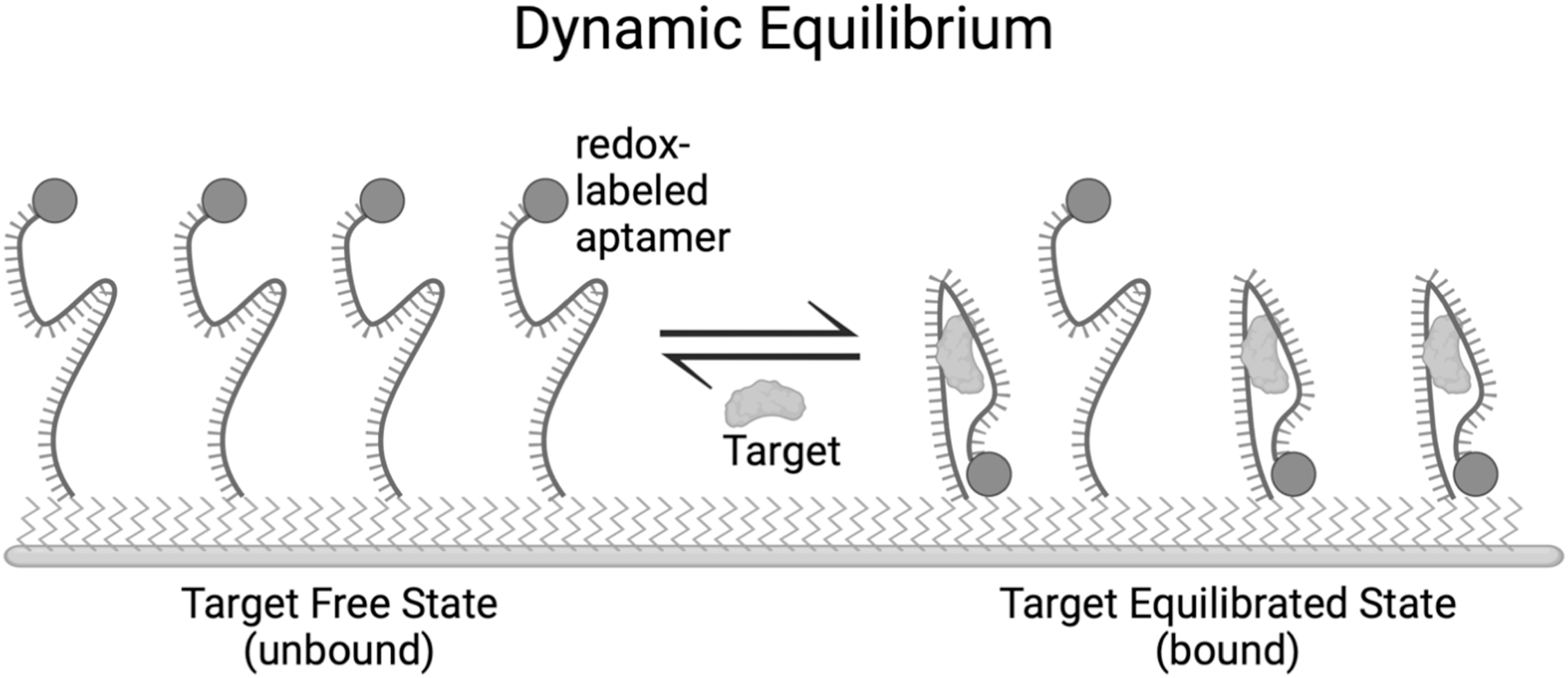
A possible scheme for aptamer target binding. The average distance between the redox reporter and the electrode surface will be different in the bound compared to the unbound state, thus resulting in a measurable change in current.

Electrochemical aptamer-based (E-AB; Fig. 1) sensors are especially useful as biosensors as they are reagentless, can perform measurements on timescales ≤2 ms,^21^ and expand the sensitivity of electrochemical measurements to targets that are not intrinsically electroactive.^22,23^ The reagentless aspect of these sensors makes them ideally suited to measure real-time changes in analyte concentration, as the output is based on conformational changes of the aptamer upon analyte binding.^24^ Selection of aptamers for specific targets is performed through a generalizable process known as systematic evolution of ligands by exponential enrichment (SELEX),^25,26^ which enables the selection of aptamers for a wide variety of targets for use in various detection schemes. Thus, E-AB sensors are adaptable to virtually any analyte. Aptamer sequences can bind with high specificity for their intended target, resulting in E-AB sensors with correspondingly high specificity and selectivity.^26–30^

## Why Focus on Electrochemical, Aptamer-Based Sensors?

We focus specifically on electrochemical, aptamer-based sensors here because they possess the ability to perform dynamic detection of a wide range of analytes coupled with the inherent sensitivity of electrochemical measurements. The overarching question is—*can E-AB sensors couple the generalizability of the aptamer-sensing mechanism, and thus measurable targets, to the demonstrated success of electroanalytical methodology in the field of chemical transmitter measurements?* This class of sensor has already made an impact in the fields of medical diagnostics,^31^ environmental analysis,^32^ food safety,^32^ and pharmacokinetics.^33^ These analytical applications benefit from the reagentless, straightforward nature of E-AB signaling.^34^ E-AB sensing platforms can be interrogated using a variety of electrochemical techniques such as square wave voltammetry (SWV), cyclic voltammetry (CV), alternating current voltammetry (ACV) and electrochemical impedance spectroscopy (EIS), providing these sensors with high sensitivity.^35^ A review of various electrochemical biosensors, including a discussion of these analysis techniques, can be found in Ref. 36.

One key attribute of E-AB sensors that makes them well suited for dynamic monitoring of small biomolecules is that they are reagentless. There is no need to apply exogenous reagents to the sample to obtain the signal. The aptamers are attached to the electrode surface, and the only requirement to obtain a measurement is that the electrodes are in contact with the sample and that the aptamer undergoes a conformation change upon target binding (see Fig. 1).^24^ The binding between aptamer and target is a reversible process which adapts to changes in solution concentrations to maintain equilibrium, and so increases in target concentration and subsequent decreases can be measured easily using E-AB sensors.^37^ The dynamic equilibrium between solution phase target concentration and aptamer-target complexes on the electrode surface is essential for applications that require dynamic measurement of target concentration such as therapeutic drug monitoring in-vitro^33^ and in-vivo.^38^

The spatial resolution of E-AB sensor measurements is determined by the length and width of the sensing electrode. E-AB sensors can be fabricated on small platforms due to the size and nature of aptamers and electrochemical signal transducers to make spatially resolved measurements, although small-sized sensors can require additional optimization efforts.^39^ While E-AB sensors seem well-positioned to tackle the analytical challenges of cellular signaling measurements, there are still specific requirements that need to be met for success of the field. In this paper, we describe advances that help position E-AB sensors so that they are ready to become a mainstay in dynamic cell signaling measurements.

## Current Status

Although there has been much research into the development of E-AB sensors and the operational mechanisms, there have been relatively few publications aimed specifically at implementing E-AB sensors for the measurement of cell signaling in real time. The measurement of cell signaling requires that the sensor be in direct contact with cell culture. One issue that comes up in this regard is that the sensing surface may become contaminated or covered with cells, which may lower or completely suppress sensor response. There have been several reports implementing cell capture using antibodies placed in a ring around the electrode, preventing direct attachment of the cells to the sensing surface. On the other hand, cells grown in collagen gels, which are directly cast onto electrode surfaces, also show responses to ATP upon cell stimulation,^40^ which indicates that the cells do not deposit onto the sensing surface to an extent that prevents measurement of the signal.

### Cytokine detection

A significant portion of the published work in this field comes from Revzin and coworkers. Their earliest work was focused on the release of interferon-*γ *(IFN-*γ*).^41^ These same sensors were later implemented to measure IFN-*γ* release in real time from CD4 T-cells, contained in a hydrogel. This device captured cells from a blood sample using antibodies. The release of IFN-*γ* could be detected from as few as 90 cells in a time frame of 15 min, resulting in a signal suppression of ∼3%. Using the antibody capture method to immobilize cells around the electrode, sensors were also developed to detect two different cytokines simultaneously.^42^ E-AB sensors were constructed with anthraquinone modified aptamers for IFN-*γ*, and methylene blue modified aptamers for tumor necrosis factor-*α* (TNF-*α*), allowing for detection of both targets from the two distinct redox peaks when using optimized surface concentrations of the two aptamers. This set-up allowed for differential signals to be observed from human T cells, which release both cytokines, as compared to U937 cells, which release only TNF-*α* upon stimulation. Measurements were taken with SWV every 20 min for 2 h, after which the sensor binding sites were saturated. Revzin and coworkers have also developed gold electrodes structured with nanowires.^43^ The nanowires on the surface enhance aptamer packing density, thus resulting in an overall current increase, and support cell intercalation between the nanowires on the surface. This construction for sensing IFN-*γ* release from CD4 T-cells resulted in the ability to detect release from as few as 300 cells ml^−1^.

Microfluidic devices have been designed for the detection of transforming growth factor *β*1 (TGF-*β*1) with E-AB sensors.^44^ These devices utilized small “cups” which could cover the sensing areas while the cells are injected into the device, protecting the sensors from biofouling. Here, the cells stuck to the collagen matrix, and therefore no antibodies were required for cell capture. After stimulation, TGF-*β*1 was monitored for 20 h, although the target reached saturating concentrations after 18 h. The rate of TGF-*β*1 production over 24 h was found to be 0.0140 pg/cell/h for stimulated cells, and 0.0009 pg/cell/h for non-stimulated cells.

### Detection of cancer markers

Pedrosa and coworkers have developed aptamer-based sensors to detect real-time release of prostate cancer biomarkers from cells. In one study, devices were constructed to detect MUC1 released from prostate cancer cells,^45^ and in a separate study similar devices were used to detect both vascular endothelial growth factor (VEGF) and prostate-specific antigen (PSA).^46^ In the case of the combined VEGF and PSA sensing platform, both aptamers were modified with methylene blue, so the detection of the two was done sequentially with separate electrodes as opposed to simultaneously. The time resolution is claimed to be less than one minute including measurement of both targets, but measurements were only taken every 20 min. Measurement of these proteins was able to differentiate between non-cancerous cells and two types of prostate cancer cells.

### ATP detection

Several sensing platforms have been used to measure ATP release from cells. The earliest example is E-AB sensors prepared on thin-film gold chip electrodes,^40^ which were used to detect ATP released by astrocytes grown in a 3D collagen matrix, allowing for cell morphology more similar to the in vivo state than 2D cultures. These sensors effectively detected ATP released from cells after stimulation with Ca^2+^, glutamate, and ionomycin, and were able to demonstrate that the various stimulants resulted in different amounts of ATP release.

Smaller scale ATP sensors were developed to detect release from single cells, via insertion into either the cytoplasm or the nucleus.^47^ The ATP concentration in the cytoplasm, nucleus, and extracellular space near the membrane were quantified under normal and glucose starvation conditions, with ATP concentrations shown to drop under glucose starvation. Additionally, Ca^2+^ stimulation resulted in the increase of ATP outside the cell due to release. Carbon fiber electrodes have also been modified to act as E-AB sensors for ATP in and around cells.^48^ Although the response time could be as low as 1 minute, it took about 5 min for the response to stabilize at 25 *μ*M ATP. The sensor could be inserted into the cytoplasm for 20 min and the nucleus for 10 min without resulting in cell death. Treatment of cells with etoposide resulted in increased ATP concentrations in all regions over 20 min of measurement, with the largest increase in the nucleus, while treatment with oligomycin resulted in decrease in ATP concentration over 3 h.

### Kanamycin detection

A small, ∼5 *μ*m diameter electrode for detection of kanamycin was recently used to measure uptake of target into salmon eggs.^49^ The real-time uptake of kanamycin from the incubation solution into salmon egg cells was measured over 6 h. The time resolution of the sensor was 2.2 ms, however, measurements were only taken every 3 min. Although this is not a measure of actual cell metabolism, it provides insight into the transfer of kanamycin across the protective cell membrane.

## Future Needs and Prospects

### Overall challenges of measuring dynamic cell signaling

To develop E-AB sensors that can be successfully used for measurements of cell signaling in real time, there are several key parameters that must be considered: (1) sensor size, (2) dynamic response characteristics (on and off rates), and 3) target detection range. Cell signaling can include a wide variety of processes that occur on various time scales (ms—h) and involve concentration changes over a large range (pM—mM), depending on the analyte of interest. The literature on altering the detection range of E-AB sensors is extensive, and many diverse methods exist to shift, widen, or narrow the dynamic response range.^50,51^ Electrochemical techniques with improved time resolution for aptamer-based sensors are also an active area of research. Additionally, if single-cell or sub-cellular measurement are desired, the size of the electrode must be adjusted to match the size of the desired sampling area. Small-sized E-AB sensors have therefore been developed for these applications. These three challenges will be discussed in more detail.

### Spatial resolution and electrode size requirements

E-AB sensors fabricated on small electrodes have proven useful in real time monitoring of metabolites and drugs in various biological specimen.^37^ Micro- or nanoelectrodes enable faster measurements by reducing the diffusion distance between the electrode surface and the target analyte point of release.^41^ This requirement is crucial for quick and real-time measurements of dynamic processes, such as neurotransmitter release at synapses.^42^ Mass transport is enhanced using small electrodes because radial diffusion becomes dominant at small electrodes as the size of the electrode decreases.^41^ The ability of small electrodes to probe the chemical environment within a short diffuse layer (defined by the experimental timescale *δ* = (*Dt*)^1/2^, where *D* is the diffusion coefficient of the target and *t* is the time of the experiment^43^) leads to this advantage. Moreover, the mass transfer coefficient (cm/s) scales roughly inversely with the electrode radius (∼D/r, where r is the electrode radius in cm).^52^ Small electrodes allow for localized measurements to be carried out in certain microenvironments, such as single cells or subcellular areas, facilitating the study of confined processes that might be missed with larger electrodes. Depending on the type of cell, the dimensions can range from a few to tens of micrometers,^44^ and sensors made for this purpose must have the necessary size resolution.

The size of sensors can be reduced while maintaining their sensitivity and specificity by fabricating micro- or nanoelectrodes using pre-existing methods and altering the electrode surface with nanomaterials.^39^ Among the work done with structure switching E-AB sensors on these small electrode platforms are reversible and reproducible E-AB sensors fabricated on 25 *μ*m modified gold electrodes to quantify analytes in undiluted serum. The authors improved sensor performance by increasing signal to noise ratio and sensitivity through electrochemical deposition of dendritic nanostructures.^53^ In the following section, considerations about the range of detectable concentrations and how this interacts with the restricted volumes of small surface area sensors will be discussed.

### Sensor dynamic range requirements

The dynamic response range of E-AB sensors is limited to 81-fold.^51^ This limited response range is due to the finite number of aptamers on the electrode surface. The binding between aptamer and target is described by the Langmuir isotherm, which predicts an 81-fold target concentration difference between 10% and 90% of aptamers bound. The actual concentration range over which detection occurs will depend on the binding affinity (K_d_) of the aptamer-target complex formation and the aptamer concentration on the electrode surface. Therefore, as discussed thoroughly in several reviews,^50,51^ the design of both the aptamer itself and the sensor must be considered to match the detection range to the relevant concentrations of the analyte that occur in biological samples. Strategies for shifting the dynamic response range while retaining the reagentless nature of the sensor include altering the aptamer sequence to obtain a different K_d_, using multiple aptamers with varying affinity on a single platform, using electrodes with nanoscale surface features, and using multi-electrode arrays.^50^ Given these considerations, the relevant biological concentration ranges of some of the common targets for real-time cell measurements must be considered when developing sensing platforms. These can range from ∼4 mM^54^ to hundreds of *μ*M^40,47,48^ for extracellular ATP and ∼10–100 pg ml^−1 55–58^ for IFN-*γ*, two common targets (see Current Status section).

Moving towards resolving single cell measurements or individual exocytosis events, the concentration range of target analyte becomes intimately linked between electrode size and distance from the site of release. Consider an ultramicroelectrode based sensor with a radius of 1 *μ*m and an insulating sheath 10 times this size. If this electrode is positioned within 0.5 *μ*m of the cell surface—a reasonable distance considering modern scanning electrochemical microscopy—we can define a volume probed by the sensors as a cylinder with a radius of 10 *μ*m and 0.5 *μ*m height. This is equal to a solution volume of 0.16 pL. The gap distance of 0.5 *μ*m versus a 20 *μ*m diameter insulating sheath means the analyte molecule will make ∼8,000 round trips per second or 0.125 ms transit time (assuming l^2^ = 2Dt, where l is the separation distance and D = 10^−5 ^cm^2 ^s^−1^ for a small molecule) compared to the 50 ms transit time to escape the gap created by the sensor, ensuring a high likelihood of finding the sensor surface. Moreover, 10,000 molecules released into that gap is equivalent to ∼100 nM concentration. Moving away from the cell surface to 10 *μ*m lowers the effective concentration to ∼5 nM. The ability to control the placement of the sensing electrode in proximity to the electrode surface is a critical experimental criterion to consider. Considering that the timeframe of diffusion to the electrode surface is so important, the sensor response time is also critical, as will be discussed.

### Sensor response time and dynamic response requirements

Compared to many techniques involving multiple reagent additions, electrochemical sensors have the potential to measure a much closer to instantaneous concentration of analyte, over a much smaller and defined spatial area. Electrochemical aptamer-based sensors have two components determining the ultimate time resolution: the response time of the sensor, and the time required to perform the electrochemical analysis. The response time of the sensor depends on the binding kinetics between the aptamer and the target, which will determine the time frame required to reach equilibrium and is also dependent on the concentration of the target. The length of electrochemical analysis varies greatly based on the technique; hence the fast time scales of pulsed voltametric techniques are popular in the field. Square-wave voltammetry and differential pulse voltammetry can be performed on time scales from a few to less than one second. Chronoamperometry has been used to interrogate E-AB sensors with a time resolution of 300 ms,^59^ and intermittent pulse amperometry (IPA) can obtain measurements with a resolution of 2 ms.^21^

Kinetics of E-AB sensor response for both tobramycin and ATP E-AB sensors have response time on the seconds timescale.^21^ The response time of the tobramycin sensor was ∼4 s, while the ATP sensor responded in ∼2 s. Current research in our laboratory indicates that this is a product of solution mixing and not true sensor response time. A surface plasmon resonance binding study of 12 aptamers for a variety of targets determined association constants in the range of 10^4^−10^5^ M^−1^ s^−1^ for many of the studied aptamers. Several aptamers, including the full-length ATP aptamer, displayed fast enough binding that the association constants could not be determined using the chosen technique.^60^

## Conclusions

As summarized in Table I, many papers that refer to real-time measurement of cell signaling do not employ measurement at especially short intervals, typically using sampling frequencies in the range of 10 to 20 min. Therefore, the speed of electrochemical analysis and kinetics of aptamer binding, relevant at short analysis times, are not of high importance in these instances. However, the development of E-AB sensors and electrochemical techniques that can be used on sub-second time scales have the potential to provide important information on faster signaling pathways like single cell vesicular exocytosis.

**Table I.** Performance characteristics of E-AB sensors used for monitoring real-time cell signaling.

Target	Sensor size	Sampling frequency	Dynamic range	References
IFN-*γ*	300 *μ*m diameter	15 min	60 pM–9 nM	61
TGF-*β*1	300 *μ*m diameter	NS (∼15 min)	1–250 ng ml^−1^	44
TNF-*α*	300 *μ*m diameter	20 min	9–88 ng ml^−1^	42
IFN- *γ*	300 *μ*m diameter	20 min	9–130 ng ml^−1^	42
IFN- *γ*	1 cm^2^	10 min fastest	0.2–100 ng ml^−1^	43
MUC1	Not specified	20 min	0.65–110 ng ml^−1^	45
VEGF	100 mm diameter	20 min	0.32–100 ng ml^−1^	46
PSA	100 mm diameter	20 min	1.1–100 ng ml^−1^	46
ATP	1 mm diameter	NS (∼5 s)	1–10 *μ*M	40
ATP	400 nm diameter × 150 nm length	5 min	0.05–2 mM	48
ATP	120 nm diameter × 120 nm length	10 min	0.059–2 mM	47
Kanamycin	2.5 *μ*m diameter	3 min	1 nM–10 *μ*M	49

NS: Not specified

In terms of size, there are many reports of fabrication of small needle-shaped sensors that allow for insertion of the sensor into the cell or cell nucleus. The technology for producing small sensors is well-developed and future work will likely focus on applying these small sensors to various cell types and aptamer targets. Selection of new aptamers for targets without known aptamers or targets for which known aptamers possess ineffective target binding for measurement in the biological concentration range will allow for expansion of E-AB sensors to new targets involved in cell signaling processes.

E-AB sensors are well suited to monitor the dynamics of small molecules in the extracellular environment. The current state of the field demonstrates that this measurement is feasible particularly in samples with large numbers of cells and relatively slow (sec-mins-hours) changes in messenger concentrations. Pioneering studies demonstrate the feasibility of the measurement, yet the questions remain as to whether E-AB sensors can be miniaturized satisfactorily to measure single and subcellular localized chemical messenger release and if the response time is suitable for capturing single exocytosis events. With this capability, we believe E-AB sensors can make significant impact in a field dominated by the world of chronoamperometry and FSCV at carbon fiber electrodes.
